# Identification of a gene, *FMP21*, whose expression levels are involved in thermotolerance in *Saccharomyces cerevisiae*

**DOI:** 10.1186/s13568-014-0067-2

**Published:** 2014-08-23

**Authors:** Toshihide Nakamura, Mami Yamamoto, Katsuichi Saito, Akira Ando, Jun Shima

**Affiliations:** 1 National Food Research Institute, National Agriculture and Food Research Organization (NARO), 2-1-12 Kannondai, Tsukuba 305-8642, Ibaraki, Japan; 2 NARO Institute of Vegetable and Tea Science, 360 Kusawa, Ano, Tsu 514-2392, Mie, Japan; 3 Ryukoku University, Fukakusa Tsukamotohon-cho, Fushimi-ku, Kyoto 612-8577, Kyoto, Japan

**Keywords:** Thermotolerance, Yeast, Gene expression, Growth rate, FMP21

## Abstract

Elucidation of the mechanism of high temperature tolerance in yeasts is important for the molecular breeding of high temperature-tolerant yeasts that can be used in bioethanol production. We identified genes whose expression is correlated with the degree of thermotolerance in *Saccharomyces cerevisiae* by DNA microarray analysis. Gene expression profiles of three *S. cerevisiae* strains showing different levels of thermotolerance were compared, and we chose three of them as candidate genes. Among these genes, *FMP21* was investigated as a thermotolerance-related gene in *S. cerevisiae* by comparing the growth at high temperature with the gene expression in eight strains. The expression ratio of *FMP21* at 37°C was correlated with the doubling time ratio at a coefficient of determination of 0.787. The potential involvement of the Fmp21 in the thermotolerance of yeasts was evaluated. The *FMP21* deletion variant showed a decreased respiratory growth rate and increased thermosensitivity. Furthermore, the overexpression of *FMP21* improved thermotolerance in yeasts. In conclusion, the function of Fmp21 is important for thermotolerance in yeasts.

## Introduction

The yeast *Saccharomyces cerevisiae* has been used in the production of traditional fermented foods and beverages as well as in the industrial production of ethanol. In the process of ethanol production, the temperature of the fermentor is raised by the heat of the fermentation, and the elevated temperature results in decreases in the cell growth, viability, and ethanol productivity of the yeast. Thermotolerant yeast is thus required for efficient ethanol production. The growth properties of *S. cerevisiae* at high temperatures differ according to the strain used, but the optimum temperature of proliferation is around 30°C. Some high-temperature-resistant strains have been isolated from tropical areas and obtained by mutational improvement, breeding, or the cell fusion of existing strains (Pasha et al. [2007]; Marullo et al. [2009]; Shi et al. [2009]). These thermotolerant yeasts were selected based on the cell growth and ethanol production at high temperature.

Analyses of genes whose expression is induced by high temperature are important for understanding the mechanisms of thermotolerance, and for the use of the genes in breeding thermotolerant yeasts. Genes related to the thermotolerance of *S. cerevisiae* have been identified by DNA microarray analyses (Eisen et al. [1998]; Sakaki et al. [2003]) and a genome-wide screening of an *S. cerevisiae* deletion mutant collection (Auesukaree et al. [2009]). Many genes were found to be important for the tolerance to heat stress (Auesukaree et al. [2009]).

Genomic regions related to the high-temperature growth of *S. cerevisiae* were identified by quantitative trait locus (QTL) mapping (Steinmetz et al. [2000]; Sinha et al. [2006], [2008]). The QTL map contained *MKT1*, *END3*, and *RHO2* as high-temperature growth quantitative trait genes. Other factors such as heat shock proteins and trehalose also function in yeast as protectants contributing to survival under heat stress conditions (Watson [1990]; Singer and Lindquist [1998]). However, the mechanisms of thermotolerance in yeasts are still unclear.

In the present study, we attempted to identify genes whose expression is correlated with the degree of thermotolerance in *S. cerevisiae*. We compared the gene expression profiles of three *S. cerevisiae* strains that showed different levels of thermotolerance. We investigated *FMP21* as a thermotolerance-related gene in *S. cerevisiae* by comparing the growth at high temperature with the gene expression in eight strains.

## Materials and methods

### Yeast strains and growth conditions

The yeasts used in this study (*Saccharomyces cerevisiae* NFRI 3122, NFRI 3145, NFRI 3155, NFRI 3205, NFRI 3225, NFRI 3236, and NFRI 3314) were obtained from the Microbiological Bank at our institute (NFRI). *Saccharomyces cerevisiae* S288C was used as a control strain. A wild-type strain, *S. cerevisiae* BY4743 and the deletion strain *fmp21*Δ derived from BY4743 were obtained from EUROSCARF (EUROpean Saccharomyces Cerevisiae ARchive for Functional analysis). The strains were grown in YPD medium (1% yeast extract [Difco Laboratory, Detroit, MI, USA], 2% peptone [Difco Laboratory], and 2% glucose) with shaking at 140 rpm.

### Determination of growth rates

The yeast strains were inoculated into YPD medium and incubated at 30°C for 24 h (pre-culture). Cell growth was monitored by measuring the optical density of the cultures at 600 nm (OD_600_) using a spectrophotometer (Ultrospec 2100 pro; GE Healthcare, Freiburg, Germany). The cells were transferred to YPD medium at a starting OD_600_ of 0.1 and incubated at 30°C with shaking. The experiments were performed in triplicate. Growth rates, expressed as the time required for the cultures to double in optical density (doubling time, *T*_d_), were calculated by comparing the optical density at multiple time points during the linear growth phase and using the following formula:

(1) $$T_{d}=\;\left(t_{2}-t_{1}\right)/\left[\log_{2}\left({\mathrm{OD}}_{2}/{\mathrm{OD}}_{1}\right)\right].$$

### Heat treatment for total RNA extraction

To confirm the heat tolerance of the strains, the cells were cultured in YPD medium under high-temperature conditions. Yeast cells were inoculated at an initial cell density of 0.1 at OD_600_ and grown to 1.0 OD_600_. Aliquots (10 mL) of exponentially growing cells were transferred to pre-heated conical flasks of replicates of each strain. The flasks were placed in a 37°C or 39°C water bath for 30 min.

### DNA microarray analysis

The experimental procedures used for the RNA extraction and DNA microarray analysis have been described previously (Nakamura et al. [2008]). The total RNA was quantified using a Bioanalyzer 2100 with an RNA 6000 Nano LabChip kit (Agilent Technologies, Palo Alto, CA, USA). Polyadenylated RNA (mRNA) from total RNA was purified using Oligotex-dT30 < Super > (Takara, Kyoto, Japan) according to the manufacturer’s instructions. We performed complementary DNA synthesis and labeling and array hybridization by following the Affymetrix user’s manual with a one-cycle target labeling and control reagent kit (Affymetrix, Santa Clara, CA, USA) with 0.4 μg of mRNA as the template material. Labeled cDNA was hybridized to Affymetrix GeneChip Yeast 2.0 arrays (Affymetrix) for 16 h at 45°C with constant rotation at 60 rpm. Washing and staining were performed using a hybridization, wash, and stain kit (Affymetrix) on a GeneChip FS-450 fluidics station (Affymetrix). Fluorescence was detected using the Affymetrix 3000 GeneArray Scanner, and the image analysis of each GeneChip was performed using the GeneChip Operating System 1.4.0 (GCOS) software from Affymetrix with the standard default settings. Probe level data based on the gene expression data from two independent experiments were imported into GeneSpring GX 11.0.2 (Agilent Technologies). Following 75th percentile shift normalization, low-intensity probes subject to high degrees of noise were removed by filtering the lowest 20% of each sample and then by filtering with flags (present and marginal). For the identification of differentially expressed genes, the Filter on Confidence function was used with a *t*-test *P*-value cutoff of 0.05, and a fold change threshold of 1.0 for the gene list was generated using the Filter on Volcano Plot tool. All experiments were done in duplicate with independently grown cells.

Microarray data from the present study have been deposited in the Gene Expression Omnibus (GEO) repository at the National Center for Biotechnology Information (NCBI) under series accession no. GSE33276.

### PCR primers and real-time RT-PCR

The oligonucleotide primer pairs listed in Table 1 were designed using Primer3 (http://primer3.sourceforge.net/). Real-time reverse transcription polymerase chain reaction (RT-PCR) was carried out using the LightCycler 2.0 instrument in glass capillaries (Roche, Indianapolis, IN, USA). Two μg of total RNA was reverse-transcribed into cDNA in a 20-μL reaction mixture using a PrimeScript Reverse Transcriptase kit (Takara). The reaction mix consisted of 5 μL standard/diluted cDNA template (1/10 to 1/1000), 0.5 μL 10× primer for the gene [final 0.5 μM primer, and 2.5–4 mM MgCl_2_], 2 μL water, and 2 μL FAST-START DNA Master SYBR Green I mix (Roche). The reaction conditions were as follows: initial denaturation at 95°C for 10 min, followed by 38 cycles of denaturation at 95°C for 10 s, annealing at 60°C for 10 s, and extension at 72°C for 10 s. The progress of real-time fluorescent PCR was monitored at 530 nm.

**Table 1:** List of primers used in this study

***Primer***	***Nucleotide sequence (5’-3’)***	***Target ORF***
Real-time PCR experiments		
YER034W-F	CACCCAGTGATAGAAAGTACCG	*YER034W*
YER034W-R	TCCTCATAGTACCTGTCCTCG	*YER034W*
prm5-F	GCAATTTTTGCGGGTTTCCTTA	*PRM5* (*YIL117C*)
prm5-R	TCGTCGCTCGATAACGGTA	*PRM5* (*YIL117C*)
YBR269C-F	TCGCAAGAGGCAATAGATCAG	*FMP21* (*YBR269C*)
YBR269C-R	TCGAATTCAGGTATGGTCTTGG	*FMP21* (*YBR269C*)
Act1-F	AGCCTTCTACGTTTCCATCC	*ACT1*
Act1-R	CTTTCAGCAGTGGTGGAGAA	*ACT1*
Construction of the *FMP21*-overexpressing strain		
URA3-F	CAGGGTCCATAAAGCTTT	
URA3-R	TTTATAAAGGCCATGAAGCT	
TDH3 pro-F	GGAAAGAAAAAGCTTCATGGCCTTTATAAAAACACGCTTTTTCAGTTC	
TDH3 pro-R	TTTGTTTGTTTATGTGTGTT	
FMP21-A	AACGCACTCAAGGTTTTTGG	
FMP21-B	AAGATGAATTGAAAAGCTTTATGGACCCTGTAGTTCTTCTTTTTATTATT	
FMP21-C	GTTTCGAATAAACACACATAAACAAACAAAATGTTGTGCGCCATCAAAAG	
FMP21-D	GTATGAATGTGCCCAGTGTA	

The PCR products were verified with both a melting curve analysis and DNA gel electrophoresis. To establish the external standard curves for the quantification of each gene, the purified PCR products were diluted in a 10-fold series (1/10 to 1/10000) and analyzed with the new assay. The expression data and associated technical errors on duplicates were calculated by using LightCycler Software 4.0 (Roche). The expression levels for each sample were normalized for the expression of *ACT1.*

The gene expression rate is based on the expression levels of the target gene versus the reference gene (*ACT1*). We confirmed that the *ACT1* expression was stable under high temperature conditions by comparing it with the expression of *ALG9*, which is considered a suitable reference gene for real-time RT-PCR (Teste et al. [2009]). The gene expression ratio is given as the ratio of the target gene expression rate after heat treatment to that in non-treated controls.

### Assay for thermotolerance

Yeast strains were precultured in YPD medium until the log phase. Then, the preculture was inoculated into 5 ml of YPD medium and shaken at 70 rpm under aerobic conditions at 30°C or 39°C. The OD_660_ was automatically recorded with an Advantec TVS062CA biophotorecorder (Advantec Toyo Co. Ltd., Tokyo, Japan).

### Construction of *FMP21*-overexpressing strains

We constructed an *FMP21*-overexpressing strain based on a previously reported method (Hasegawa et al. [2012]) using the primer set listed in Table 1. Briefly, the promoter region of the glyceraldehyde-3-phosphate dehydrogenase gene (*TDH3*), which allows constitutive expression at a high level, was fused with the *URA3* marker gene and then inserted upstream of the start codon of the *FMP21* gene in BY4743. The resultant *FMP21*-overexpressing strains (*MATa/α ura3Δ0 URA3-P*_*TDH3*_*-FMP21*) and control strains (*MATa/α ura3Δ0 URA3*) were analyzed for *FMP21* expression levels and thermotolerance. The expression levels of *FMP21* were determined by real-time RT-PCR as described above.

## Results

### Screening of thermotolerance-related genes by DNA microarray analysis

**Figure 1:**
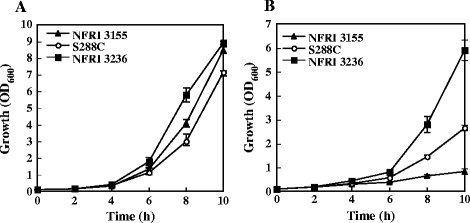
**Effect of temperature on the growth of three yeast strains (NFRI 3236, NFRI 3155 and S288C).** Each strain was grown in YPD with shaking at 30°C **(A)**, or 39°C **(B)**.

We used three yeast strains: a thermotolerant strain, NFRI 3236; a thermosensitive strain, NFRI 3155; and a standard strain, S288C (Figure 1). The doubling times (T_d_) of NFRI 3236, NFRI 3155 and S288C at 39°C were shown to be 1.75, 2.90 and 2.11 h, respectively. On the other hand, the T_d_ values of NFRI 3236, NFRI 3155 and S288C at 30°C, a standard growth condition for the strains, showed no significant differences and were 1.15, 1.28 and 1.34 h, respectively.

We performed DNA microarray experiments in order to compare gene expression responses to the heat treatment at 39°C with the gene expressions in non-treated control cells. We first selected genes (441 genes) that showed a higher than 1.5-fold change in their expression in both NFRI 3236 and S288C, and then selected genes that showed a > 1.5 ratio of fold-change values of NFRI 3236 to S288C or of S288C to NFRI 3155. We then selected three genes whose expression ratios were correlated with the level of thermotolerance in the three strains (Table 2). The fold-change values of *YER034W*, *YIL117C* (*PRM5*) and *YBR269C* (*FMP21*) were more clearly correlated with the rank order of thermotolerance. We thus chose *YER034W*, *PRM5* and *FMP21* as candidate thermotolerance-related genes.

**Table 2:** Selected genes and their expression values in DNA microarray analysis

**Gene**	**Fold change**			**Descriptions of gene products**
	**NFRI3236**	**S288C**	**NFRI3155**	
*FMP21*	3.68	2.18	1.35	Putative protein of unknown function; the authentic, non-tagged protein is detected in highly purified mitochondria in high-throughput studies
*YER034W*	3.10	1.51	0.49	Putative protein of unknown function; non-essential gene; expression induced upon calcium shortage
*PRM5*	48.21	23.05	15.07	Pheromone-regulated protein, predicted to have 1 transmembrane segment; induced during cell integrity signaling

### Expression analysis of the three candidates by real-time RT-PCR

**Figure 2:**
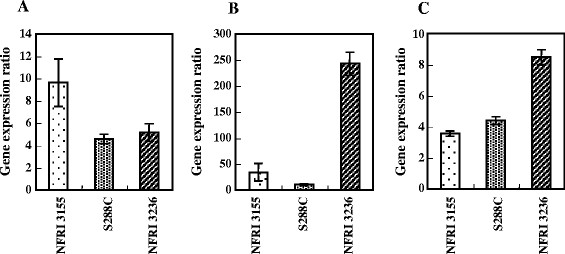
**Comparison of the gene expression ratios of the selected genes. (A)***YER034W*, **(B)***PRM5*, and **(C)***FMP21*. Bars indicate the mean ratios for two independent biological replicates. Error bars = standard deviation.

We verified the expression of *YER034W*, *PRM5* and *FMP21* in NFRI 3236, NFRI 3155 and S288C under heat stress conditions. The expression ratio (i.e., the expression level at high temperature/the expression level at 30°C) of each candidate gene was measured by real-time RT-PCR (Figure 2). The *ACT1* gene was used as the expression standard. The expression ratio of *FMP21* was 3.47 in NFRI 3155, 4.50 in S288C and 8.50 in NFRI 3236, and it increased (statistically significant at *P* <0.05) according to the thermotolerance of the yeast strains. However, the expression ratios of *YER034W* and *PRM5* showed no relationship with the thermotolerance of the yeast strains.

### Relationship between the expression of *FMP21* and the thermotolerance of yeast strains

**Figure 3:**
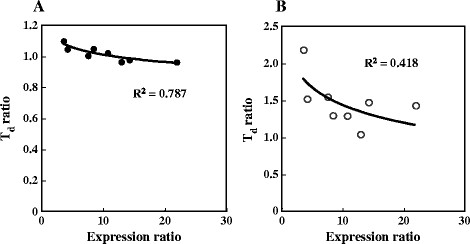
**Relationship between the expression ratio of** ***FMP21*** **and the doubling time (T**_**d**_**) ratio at 37°C (30°C/37°C) (A) and at 39°C (30°C/39°C) (B).** The coefficients of determination (R^2^) were 0.787 **(A)** and 0.418 **(B)**.

To assess the relationship between the expression of the *FMP21* gene and the thermotolerance of yeast strains, we measured the expression levels at 30°C, 37°C, and 39°C in five strains (NFRI 3122, NFRI 3145, NFRI 3205, NFRI 3225, and NFRI 3314) in addition to the three strains described above (eight strains total). Figure 3 is a graph plotting the expression ratios of *FMP21* against the T_d_ ratio. With the heat treatment of 37°C, the increase of the expression ratio of *FMP21* by heat treatment was correlated with the T_d_ ratio at a coefficient of determination of 0.787. In contrast, there was weak correlation at 39°C (coefficient of determination, 0.418).

### Disruption and overexpression of the *FMP21* gene

**Figure 4:**
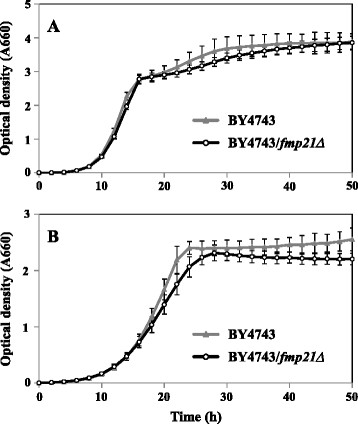
**Growth of the wild-type (BY4743) and** ***fmp21Δ*** **strains under high temperature.** Both strains were grown in YPD with shaking at 30°C **(A)** or 39°C **(B)**. Three experiments were repeated to ensure reproducibility and gave equivalent results. Each plot represents the average of triplicates.

To determine whether Fmp21 plays a role in the response to heat stress, we examined the growth ability of the *FMP21* deletion strain (*fmp21Δ*) in YPD medium at 39°C (Figure 4). The growth ability of *fmp21Δ* in YPD medium at 30°C was nearly the same as that of the wild-type strain; however, there was a reduction in the growth rate of *fmp21Δ* in the late growth phase. Under the high temperature condition, *fmp21Δ* showed a slower growth rate than the wild-type strain in the log-phase. This result revealed that the deletion of *FMP21* increased the sensitivity to high temperature.

**Figure 5:**
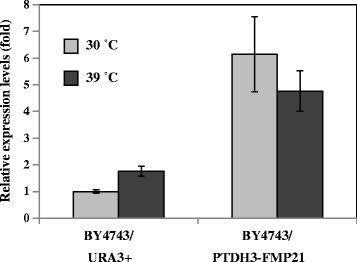
**Transcriptional analysis of** ***FMP21*** **in the** ***FMP21*-overexpressing strains.** The expression data of the *FMP21*-overexpressing strains were compared with those of the control strains. The values are the means of results from three independent strains.

**Figure 6:**
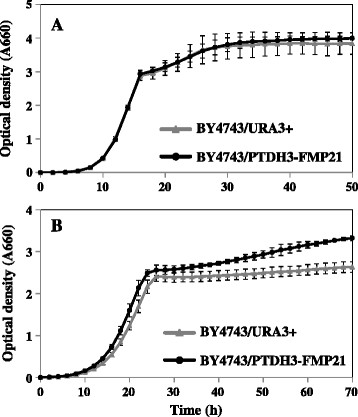
**Growth of the** ***FMP21*-overexpressing strains under high temperature.** Both the *FMP21*-overexpressing strains and the control strains were grown in YPD with shaking at 30°C **(A)** or 39°C **(B)**. The values are the means of results from three independent strains.

To evaluate the phenotype of *FMP21* overexpression, we constructed *FMP21*-overexpressing strains and compared their thermotolerance with that of the control strains. *FMP21* was overexpressed using promoter sequences of the highly expressed *TDH3* gene. Then we confirmed the mRNA expression of *FMP21* by real-time RT-PCR (Figure 5). In the *FMP21*-overexpressing strains (BY4743/PTDH3-FMP21), the transcriptional expression level of *FMP21* was approximately 6-fold higher under non-stress conditions and was approximately 3-fold higher at 39°C (the differences are statistically significant at *P* <0.01). Overexpression of *FMP21* had no effect on the growth rate at 30°C (Figure 6). The growth rate of *FMP21*-overexpressing strains was higher than that of the control strains when the cells were incubated at 39°C (Figure 6). These results suggested that yeast strains with enhanced *FMP21* expression exhibited the improved thermotolerance.

## Discussion

We found a correlative increase of *FMP21* expression along with the increase in growth rate at high temperature in yeast strains. The results of our DNA microarray analysis using three strains showed increased expression ratios under heat conditions for *FMP21*, *YER034W* and *PRM5*. However, in the verification test, the expression rate of *YER034W* showed the lowest correlation among the three genes. In addition, the expression rate of *PRM5* was significantly increased by heat treatment only in the NFRI3236 strain. The expression levels in some of the genes were very different among the yeast strains, which had different genetic backgrounds. *PRM5* is also one of the genes whose expression strongly depends on the genetic backgrounds of the strains.

The expression ratio of *FMP21* induced by heat treatment was significantly correlated with the growth ratio at 37°C. However, there was a weak correlation at 39°C. Generally, *S. cerevisiae* strains show various levels of thermotolerance depending on their genetic backgrounds. The maximum temperature for growth is under about 40°C. The strains used in this study grew unstable at 39°C, and there is therefore a high possibility that the gene expressions at 39°C would also be unstable. Therefore, we speculated that the unstable gene expressions at 39°C may have been responsible for the relatively low value of the coefficient of determination.

In yeasts, Hsf1 and Msn2/4 are major transcription factors to respond to heat stress. Hsf1 and Msn2/4 bind to heat shock elements (HSEs) and stress response elements (STREs), respectively, in the promoter region of heat shock proteins and activate the expression of target gene transcription (Morano et al. [2012]). Although the expression of *FMP21* was induced by heat stress, we did not find the typical consensus sequences of HSEs and STREs in the promoter region of *FMP21*. The *FMP21* expression may be controlled by unknown mechanisms. In contrast to the expression pattern of *FMP21*, Sakaki et al. ([2003]) reported that the expressions of mitochondrial genes were repressed in yeast cells grown at 37°C. We are interested in the functions of Fmp21—specifically whether this protein is involved in the repression of mitochondrial genes in 37°C cultivation.

*FMP21* encodes a protein localized in mitochondria, but its function is still unknown (Reinders et al. [2006]). Steinmetz et al. ([2002]) reported that the *FMP21* deletion variant showed a decreased rate of respiratory growth. We also confirmed that the *fmp21Δ* strains showed a decreased growth rate in medium containing glycerol as a sole carbon source (data not shown). The *fmp21Δ* strains showed a decreased growth rate after diauxic shift, suggesting that one of the functions of Fmp21 is to contribute to the respiratory metabolism in mitochondria of yeasts. After diauxic shift, yeasts are stressed by the lack of nutrients and by the accumulation of toxic metabolites such as ethanol. Outten et al. ([2005]) reported that the *FMP21* deletion variant was hyperoxia-sensitive. The absence of Fmp21 may render the mitochondrial function more stress-sensitive.

The *FMP21* deletion variant was a thermosensitive strain. As growth delay was observed in the late log phase, *fmp21Δ* strains might be sensitive to ethanol under high temperature conditions. However, the sensitivity to 5% (v/v) ethanol of *fmp21Δ* strains was similar to that of the wild-type strain at 30°C (data not shown). Thus it is unlikely that a function of Fmp21 is involved in ethanol tolerance. We therefore consider that the function of Fmp21 is important for the thermotolerance of yeasts.

The increased accumulation of thermoprotectants in yeasts occasionally leads to increased thermotolerance. The overexpression of heat shock proteins and the enzyme for trehalose synthesis have been shown to successfully enhance the thermotolerance of yeasts (Cheng et al. [1992]; An et al. [2011]). In this study, the *FMP21* gene was overexpressed using the promoter region of the *TDH3* gene. Although the expression of *TDH3* was decreased under high temperature (based on the DNA microarray analysis data), the expression of *FMP21* in the *FMP21*-overexpressing strains was higher than that in the control strains. The overexpression of the *FMP21* gene enhanced the thermotolerance of yeasts in aerobic cultures. As the overexpression of the *FMP21* gene also enhanced the growth in static cultures under high temperature conditions, there was no significant difference in ethanol productivity (data not shown). These results suggest that Fmp21 contributes to thermotolerance of yeasts, and that it may protect mitochondria against high temperature-related damages.

Thermotolerant yeast strains are still required in bio-industries. Various approaches have been adopted in an attempt to improve the thermotolerance of yeast strains, including the use of mating, fusion or genetic hybridization (Pasha et al. [2007]; Marullo et al. [2009]; Shi et al. [2009]). However, these approaches were based on stochasticity and used various yeast strains with different genetic backgrounds. It is thus difficult to draw conclusions from these studies that would help in the acquisition of more thermotolerant strains. The results presented here are applicable to various strains that have different genetic backgrounds. It may also be possible to use these results to quantify and evaluate thermotolerance, and for the systematic construction of more thermotolerant strains.

## Competing interests

The authors declare that they have no competing interests.
